# Behavioral and emotional adverse events of drugs frequently used in the treatment of bipolar disorders: clinical and theoretical implications

**DOI:** 10.1186/s40345-016-0047-3

**Published:** 2016-02-16

**Authors:** Alejandro Szmulewicz, Cecilia Samamé, Pablo Caravotta, Diego J. Martino, Ana Igoa, Diego Hidalgo-Mazzei, Francesc Colom, Sergio A. Strejilevich

**Affiliations:** Bipolar Disorder Program, Neurosciences Institute, Favaloro University, Buenos Aires, Argentina; National Scientific and Technical Research Council (CONICET), Buenos Aires, Argentina; Bipolar Disorders Program, IDIBAPS, CIBERSAM, Barcelona, Catalonia Spain; Hospital de Emergencias Psiquiátricas Torcuato de Alvear (HEPTA), Buenos Aires, Argentina; Congreso 2477 Dto. D (1428), Buenos Aires, Argentina

**Keywords:** Bipolar disorder, Cognition, Behavioral adverse events, Emotional detachment, Apathy

## Abstract

**Background:**

Behavioral and emotional adverse events induced by drugs commonly prescribed to patients with bipolar disorders are of paramount importance to clinical practice and research. However, no reviews on the topic have been published so far.

**Methods:**

An extensive search was performed. Reports were reviewed if they described behavioral side effects related to pharmacological treatments for bipolar disorders in healthy subjects or patients with different neuropsychiatric disorders. For this review, lithium, antipsychotics, anticonvulsants and selective serotonin reuptake inhibitors were included.

**Results:**

Apathy or emotional blunting, diminished sexual desire, and inability to cry were reported to be associated with exposure to selective serotonin reuptake inhibitors. Neuroleptic-induced deficit syndrome/emotional detachment and obsessive–compulsive symptomatology and decision-making modifications. A lithium-related amotivational syndrome was also reported in the literature. Furthermore, hypersexuality and obsessive–compulsive symptoms have been noted in subjects treated with lamotrigine.

**Limitations:**

Primary studies on drug-related adverse events are scant so far and most of the data currently available derive from case reports. Moreover, most of the evidence reviewed is based on studies performed on healthy subjects and patients with neuropsychiatric conditions other than bipolar disorders.

**Discussion:**

There is a remarkable dearth of data on behavioral adverse events of pharmacological treatment for bipolar disorders. However, the pieces of evidence available at present, though scant and scattered, suggest that different behavioral adverse events may be related to pharmacological treatment for these disorders. The implications of these findings for research and management of patients with mood disorders are discussed.

## Background

Behavioral adverse events (BAEs) induced by pharmacological treatment are defined as symptoms referable to the central nervous system that produce a characteristic pattern of change in behavior and/or emotional response that is temporally related to the administration of a given drug, which exerts its effect alone or in combination with other drugs (Gates 2000). These symptoms can involve emotional, social, hedonistic, and/or sexual features and range from changes in normal behavior, such as sexual interest, to the emergence of complex behavior as, for example, pathological gambling. BAEs can be considered as unwanted events, but also as part of the therapeutic effect of drugs in some cases. A proper knowledge of BAEs is indispensable to achieve an adequate management of psychiatric conditions. Some BAEs are strong predictors of treatment adherence and can sometimes be quite similar to illness symptomatology, causing, due to these overlaps, problems in diagnosis and clinical assessment (Awad et al. 1996). Furthermore, studying BAEs may provide a pathway into the physiology of both normal and abnormal behavior, and into the mechanism of action of psychotropic drugs (Strejilevich et al. 2012).

Despite the clinical and theoretical relevance of BAEs, research and evidence-based descriptions on these topics are scarce and their consideration in clinical practice is almost null. Antipsychotic-related BAEs are an excellent way to describe this circumstance. Actually, the serendipitous discovery of antipsychotics was due to the initial description of the BAEs produced by these drugs. Laborit and Huguenard (1951) as well as Delay and colleagues (Delay et al. 1952) began to experimentally use chlorpromazine (the former as a pre-anesthetic and the latter as treatment for psychotic agitation) when they heard of a substance that produced affective indifference and lack of initiative both in experimental animals and in healthy volunteers. The term “neuroleptics” was then coined to name these drugs, underlining their ability to produce psychomotor retardation, affective indifference, and lack of initiative, which were considered their primary mechanisms of action (Deniker 1983; López-Muñoz et al. 2005a, b). However, concomitantly to the expanding use of neuroleptics, physicians began to set aside this way to understand their clinical effects at the same time that the term “antipsychotic” was gradually extended as a way to name these drugs (King and Voruganti 2002). Around the 1970s, the interest in neuroleptic BAEs reemerged. In a series of original investigations, van Putten and colleagues showed that both motor and BAEs of antipsychotics helped to explain why many patients were reluctant to take these drugs and developed useful tools for early detection of patients who would not be adherent to treatment (Van Putten 1974, 1975; Van Putten and May 1978; Van Putten et al. 1981). However, these concepts were never fully incorporated into clinical practice or research.

In the 1990s, when atypical antipsychotics were launched onto the market, antipsychotic BAEs gained new attention (Voruganti et al. 1999; Naber et al. 2001; Chue 2006; Nasrallah and Lasser 2006). Newer terminology and tools for assessment began to appear, and an association between these BAEs and D2 and D1 receptor occupancy was described, which was made possible by neuroimaging techniques (Lewander 1994; Naber 1995; De Haan et al. 2004, 2005; Lataster et al. 2011; Takeuchi et al. 2013). Lastly, fifty years after the discovery of antipsychotics, the capacity of these compounds to produce affective indifference regained a central position in a model proposed by Kapur (2003), in which the effects of these drugs at the behavioral and cellular levels were integrated in a solid framework for the first time. This model returns over the observations of the pioneers in the use of antipsychotics, proposing that these drugs do not extinguish psychotic symptoms, but rather, they produce emotional detachment due to down-regulation of dopamine turn-over. It also assumes that apathy and lack of initiative is an unwanted consequence of the same psychological mechanism that relieves psychotic symptoms (Kapur et al. 2006). However, leaving aside the progress and setbacks of research on antipsychotics, the underestimation of such secondary events has remained constant in both research and clinical settings.

Remarkably, although bipolar disorders (BDs) are more prevalent than schizophrenia and their treatment usually involves a larger variety of psychotropic drugs, no reviews of BAEs in the treatment of these disorders have been published. The aim of this work is to provide an updated overview of BAEs associated with those drugs more frequently used in the treatment of BDs and to discuss clinical and theoretical implications of research findings.

## Methods

For the purpose or the current review, reports documenting the presence of BAEs in neuropsychiatric disorders or healthy control subjects were considered. We took into consideration only those drugs recommended as main treatment options for BDs by current clinical guidelines (Grunze et al. 2013; Yatham et al. 2013). Online databases (MEDLINE, SCOPUS, EMBASE and ClinicalTrials.gov) were searched using the following terms: “SSRIs” or “MOOD STABILIZERS” or “ANTICONVULSIVANTS” or “LITHIUM” or “ANTIPSYCHOTICS” or “NEUROLEPTICS” cross referenced with “SAFETY” or “TOLERABILITY” or “SIDE/ADVERSE EFFECTS” or “BEHAVIOR” or “CONDUCT.” We restricted our search parameters to reports published in English or Spanish up to August 2015. The reference lists of the articles selected for inclusion were also searched for relevant reports. With the objective to put focus on behavioral side effects, information on neurocognitive (attention, memory, processing speed, etc.), motor (akathisia, parkinsonism, etc.), and autonomic (erectile dysfunction, ejaculatory delay, anorgasmia, etc.) adverse events was excluded. Neither were considered side effects on vigilance and appetite regulation. In addition, since it is not clear whether switch to mania or impulsivity due to SSRI treatment is a behavioral adverse event or a modification in the course of illness, information on this issue was excluded. Likewise, symptoms related to the underlying neuropsychiatric condition (irritability, lethargy or fatigue) were excluded. Finally, we did not review reports on patients with mental retardation since this population may be unable to express discomfort from adverse events except by a change in their behavior (Gates 2000). After identifying the most prevalent BAEs for each drug, data were summarized in a narrative fashion.

## Results

A summary of the evidence for BAE induced by the main pharmacological treatments for BDs is presented in Table 1.

**Table 1:** Quality of evidence (parenthesis) and neuropsychiatric conditions in which BAEs were described (brackets)

	SSRIs	Lithium	AP	LMT
Apathy—emotional blunting	Arnone et al. (2009) *(E) [HV]* Barnhart et al. (2004) *(C) [MDD; AD]* Bolling and Kohlenberg (2004) *(D) [MDD]* Di Giannantonio and Martinotti (2012) *(D) [MDD]* Fava et al. (2006) *(D) [MDD]* Garland and Baerg (2001) *(F) [MDD; AD]* George and Trimble (1992) *(F) [AD]* Harmer et al. ( 2003, 2004, 2008, 2011) *(E) [HV]* Harmer et al. (2009) *(D) [MDD]* Hoehn-Saric et al. (1990, 1991) **(** *F) [AD]* McCabe et al. (2010) *(E) [HV]* Opbroek et al. (2002) *(D) [MDD]* Padala et al. (2012) *(F) [MDD; AD]* Popovic et al. (2015) *(D) [MDD; AD]* Price et al. (2009) *(D) [MDD; AD]* Raskin et al. (2012) *(B) [MDD]* Rawlings et al. (2010) *(E) [HV]* Reinblatt and Riddle (2006) *(D) [AD]* Rothschild et al. (2014) *(D) [MDD]* Wongpakaran et al. (2007) *(D) [MDD]*	Belmaker et al. (1979) *(D) [BD]* Bonetti et al. (1977) *(D) [BD; MDD]* Folstein et al. (1982) *(D) [BD]* Judd et al. (1977) *(E) [HV]* Kropf and Muller-Oerlinghausen (Kropf and Müller-Oerlinghausen 1975; Kropf and Muller-Oerlinghausen 1979 *) (E) [HV]* White et al. (1979) *(E) [HV]*	Awad and Voruganti (2004) **(** *D) [SZ]* Awad et al. (1996) *(D) [SZ]* Belmaker and Wald (1977) *(E) [HV]* deHaan et al. (de Haan et al. 2000; De Haan et al. 2005) *(D) [SZ]* deHaan et al. (2003) *(B) [SZ]* Fischel et al. (2013) *(D) [SZ]* Gervin et al. (1999) *(D) [SZ]* Kim and Byun (2010) *(D) [SZ]* Lambert et al. (2003) *(D) [SZ]* Lataster et al. (2011) *(D) [SZ]* Lewander (1994) *(D) [SZ]* Moncrieff et al. (2009) *(D) [SZ; BD]* Takeuchi et al. (2013) *(D) [SZ]*	N/A
Decision-making impairment	Cools et al. (2008) *(E) [HV]* Crockett et al. (2012) *(E) [HV]* Macoveanu et al. (2014) *(E) [HV]* Molero et al. (2015) *(D) [MDD; BD; AD; SZ]* Price et al. (2009) *(D) [MDD]* Rogers et al. (1999) *(E) [HV]* Rogers et al. (2003) *(E) [HV]* Tanaka et al. (2009) *(E) [HV]*	N/A	Adida et al. (2011) *(D) [SZ]* Heerey et al. (2008) *(D) [SZ]* Hutton et al. (2002) *(D) [SZ]* Poletti et al. (2011) *(D) [PD]* Prentice et al. (2005) *(D) [SZ]* Sevy et al. (2006) *(E) [HV]* Trémeau et al. (2008) *(D) [SZ]* Vrshek-Schallhorn et al. (2013) *(E) [HV]*	N/A
Diminished libido	Atmaca et al. (2011) *(D) [MDD]* Aursnes and Gjertsen (2008) *(A) [MDD]* Clayton and Montejo (2006) *(D) [MDD]* Clayton et al. (2002) *(D) [MDD]* Dueñas et al. (2011) *(D) [MDD]* Gittlin et al. (2002) *(D) [MDD]* Kennedy et al. (2008) *(B) [MDD]* Montejo et al. (2001) *(D) [MDD]* Opbroek et al. (2002) *(D) [MDD]* Serretti and Chiesa (2009) *(D) [MDD]*	N/A	N/A	N/A
Obsessive–compulsive symptoms	N/A	N/A	DeVylder et al. (2012) *(D) [SZ]* Hagen et al. (2013) *(D) [SZ]* Jonkers and deHaan (2002) *(F) [BD]* Kapur et al. (1998, 1999, 2006) *(D) [SZ]* Lemke and Bustillo (2013) *(F) [BD]* Lin et al. (2006) *(D) [SZ]* Mahendran et al. (2007) *(D) [SZ]* Niendam et al. (2009) *(D) [SZ]* Schirmbeck and Zink (2012, 2013) *(D) [SZ]* Schirmbeck et al. (2011 2013) *(D) [SZ]* Stamouli and Lykouras (2006) *(F) [MDD; BD; SZ]* Szmulewicz et al. (2015a, b) *(D) [SZ]*	Alkin et al. (2007) *(F) [BD]* Kemp et al. (2007) *(F) [BD]* Kuloglu et al. (2009) *(F) [BD]* Lee et al. (2011) *(D) [E]* Lombroso (1999) *(F) [TS]* Seemüller et al. (2006) *(F) [BD]* Szmulewicz et al. (2015b) *(D) [SZ]* Verma et al. (1999) *(F) [E]*
Inability to cry	Oleshansky and Labbate (1996) *(F) [MDD; AD]* Opbroek et al. (2002) *(D) [MDD]* Holguin-Lew and Bell (2013) *(F)* *[MDD; AD]*	N/A	N/A	N/A
Self-injuries	Price et al. (2009) *(D) [MDD]* Miller et al. (2014) *(D) [MDD]*	N/A	N/A	N/A
Hypersexuality	N/A	N/A	N/A	Grabowska-Grzyb et al. (2006) *(F) [E]*

*MDD* major depressive disorder, *AD* anxiety disorders, *BD* bipolar disorder, *SZ* schizophrenia, *PD* Parkinson’s disease, *E* epilepsy, *TS* Tourette’s Syndrome, *HV* healthy volunteer

Quality of evidence

*A* Meta-analysis of randomized controlled trials

*B* At least one randomized, controlled, double-blinded study

*C* Systematic review of studies

*D* Cohort studies/open or non-randomized studies/observational studies in patient sample/Narrative review

*E* Healthy volunteers studies

*F* Case report/Case series/Case control studies

*N/A* Not available

### Selective Serotonin Reuptake Inhibitors (SSRIs)

Four BAEs associated with SSRIs use were identified: apathy or emotional blunting, inability to cry, diminished sexual desire, and decision-making modifications.

#### Apathy or emotional blunting

Since the launch of SSRIs, evidence about behavioral changes exceeding the therapeutic effect of these drugs began to appear. In a book regarded as the landmark work about antidepressants, Kramer (1993) reported behavioral and personality changes in patients treated with fluoxetine. Although some of these changes may be accounted for by hypomanic symptoms, others can clearly be regarded as apathy or emotional blunting induced by this SSRI. From then on, data from different sources have documented the capacity of these drugs to ‘attenuate’ or ‘set aside everyday concerns,’ beyond their effect on depressive symptoms. A phenomenological description of this BAE was provided by a qualitative study based on semi-structured individual interviews performed to 38 patients treated with SSRIs due to depressive or anxiety disorders (Price et al. 2009). This investigation found that subjects experienced varying degrees of emotional detachment, which ranged from feeling as ‘just not caring’ about things previously considered as important to complete emotional numbing. Some participants felt like ‘covering up’ who they really were and reported financial and working problems because of ‘just not caring.’ This detachment was experienced as a beneficial effect by some patients, but others experience it as a decrease in normal emotional responsiveness.

The frequency of apathy/emotional blunting occurrence during treatment with SSRIs has not been consistently established. Reports vary widely, ranging from 20 (Bolling and Kohlenberg 2004) to 80 % of patients receiving these antidepressants (Opbroek et al. 2002). Apathy-emotional blunting could appear independently of the condition for which the SSRI is prescribed (major depressive disorder or anxiety disorders) (Barnhart et al. 2004) and has been found in young adults and adolescents (Hoehn-Saric et al. 1990, 1991; George and Trimble 1992), older adults (Wongpakaran et al. 2007), and pediatric population (Garland and Baerg 2001; Reinblatt and Riddle 2006) with depression or anxiety disorders. Emotional blunting during treatment with SSRIs in unipolar depression might be independent of the therapeutic effect of these drugs and could appear even after remission is achieved (Fava et al. 2006; Popovic et al. 2015).

Although relatively little or no research on the functional impact of apathy-emotional blunting has been conducted so far, some reports suggest that the emergence of this BAE could have a negative impact on normal functioning (Barnhart et al. 2004; Price et al. 2009; Padala et al. 2012; Rothschild et al. 2014).

Clinical studies have brought support to the specificity of SSRIs to cause apathy-emotional blunting (Wongpakaran et al. 2007; Di Giannantonio and Martinotti 2012) and, more specifically, to the association between these BAEs and 5HT2C agonism (Gobert et al. 2002; Arnone et al. 2009; Harmer et al. 2011). SSRI-induced apathy does not revert after treatment with a noradrenergic and serotoninergic reuptake inhibitor (Raskin et al. 2012). The chronic elevation of serotonin levels in the nucleus accumbens leads, due to 5HT2C agonism, to a down-regulation of dopamine turn-over in circuits consistently associated with apathy or emotional blunting (Levy and Dubois 2006; Stahl 2013). A series of studies using emotional cognition paradigms have shown that SSRI antidepressants produce changes in emotional processing modifying the recognition of all basic emotions such as happiness, sadness, fear, disgust, and surprise (Browning et al. 2007; Harmer et al. 2003, 2004, 2008, 2011) in healthy volunteers and depressive subjects (Harmer et al. 2009). In contrast, other antidepressants with a different mechanism of action such as reboxetine (Harmer et al. 2004), mirtazapine (Arnone et al. 2009; Rawlings et al. 2010) or agomelatine (Harmer et al. 2011) may modify the processing of happiness and sadness not modifying other basic emotions. In a recent study performed on 45 healthy volunteers, citalopram diminished neural processing of both rewarding and aversive stimuli while reboxetine did not suppress activity following reward stimuli and only mildly suppressed activity during the aversive conditions (McCabe et al. 2010).

*Findings in BDs*: Although this BAE has been studied in depression, we have not found data from patients with BDs.

#### Inability to cry

SSRI treatment has been associated with inability to cry in situations that would normally elicit crying (Oleshansky and Labbate 1996; Opbroek et al. 2002). Although this event was considered as part of the SSRI-related emotional blunting/apathy complex, evidence from a case report of seven patients on SSRIs presenting with sudden inability to cry but without concomitant apathy criteria suggested that these BAEs might be independent (Holguin-Lew and Bell 2013).

*Findings in BDs*: we have not found data from patients with BDs.

#### Diminished sexual desire

SSRI-induced sexual dysfunction includes modifications in every stage of normal sexual functioning. The prevalence of this BAE was estimated around 20–45 %, being diminished libido the most frequent event (Aursnes and Gjertsen 2008; Clayton et al. 2002). Sexual dysfunction might be present independently of the condition for which these drugs are prescribed (Montejo et al. 2001).

SSRI antidepressants diminish sexual interest, while those compounds with noradrenergic and dopaminergic reuptake inhibitor properties lack this effect (Serretti and Chiesa 2009; Clayton and Montejo 2006). SSRI-induced diminished libido does not revert after the addition of a noradrenergic reuptake inhibitor while it does after the addition of a dopaminergic and noradrenergic agents like mirtazapine, bupropion, or agomelatine (Gitlin et al. 2002; Kennedy et al. 2008; Atmaca et al. 2011; Dueñas et al. 2011). This suggests that SSRIs cause this effect by impairing normal dopaminergic transmission, which is physiologically connected with the emotional blunting mechanism (Bijlsma et al. 2014). In this line, it has been proposed that diminished libido induced by SSRIs may be a sexual concomitant of emotional blunting. Indeed, Opbroek et al. (2002) found that 80 % of a sample of 15 patients treated with SSRIs experienced diminished libido and also met criteria for apathy, inability to cry, and diminished creativity. None of the patients had significant depressive symptomatology at the time of the evaluation. Furthermore, there was no significant correlation between emotional blunting scores and depressive scores measured with the HAMD scale.

*Findings in BDs*: we have not found data from patients with BDs.

#### Decision-making modifications

Although some ecological studies and subjective reports were published on possible changes or difficulties in decision-making processes in persons treated with SSRI (Kramer 1993; Price et al. 2009; Molero et al. 2015), research on this topic is scarce. In a recent study (Macoveanu et al. 2014), 29 healthy volunteers were exposed to 40 mg fluoxetine for three weeks finding a significant reduction in neural responses to risky choices in orbitofrontal cortex during a gambling task. The authors suggested that these changes in decision-making might be pathophysiologically related to emotional blunting processes. The functional impact of this possible BAE remains understudied, although some authors have related changes in risk-taking with the increase in violence, crimes and self-injuries reported in young patients treated with SSRIs and other related antidepressants (Molero et al. 2015; Miller et al. 2014).

Serotonin has long been implicated in decision-making processes. It has been suggested that serotonin is involved in processing and avoiding negative outcomes (Rogers et al. 2003; Tanaka et al. 2009), predicting future punishment (Crockett et al. 2012), and loss aversion (Cools et al. 2008). A significant reduction in the probability to choose the most likely outcome and a switch toward making the risky choice was found in healthy volunteers exposed to serotonin depletion (Rogers et al. 1999; Long et al. 2009).

*Findings in BDs*: we have not found data from patients with BDs.

### Antipsychotics (APs)

Different BAEs due to APs were found and subsumed, for the purposes of their description, into three subgroups: neuroleptic-induced deficit syndrome/emotional detachment; obsessive–compulsive symptomatology; and decision-making modifications.

#### Neuroleptic-induced deficit syndrome (NIDS)/emotional detachment

Many studies performed on healthy volunteers and clinical populations show that exposure to APs produces a syndrome of dysphoria-apathy-apragmatism and loss of creativity. This syndrome has received different names (*“neuroleptic dysphoria,” “akinetic depression,” “neuroleptic*-*induced deficit syndrome”,* among others).

A variety of phenomenological descriptions of this syndrome are available, including systematic descriptions (Lewander 1994; Awad et al. 1996), self-reports from patients (Moncrieff et al. 2009) and healthy volunteers studies (de Haan et al. 2005) even of psychiatrists who voluntarily exposed themselves to these drugs (Belmaker and Wald 1977). The behavioral and emotional adverse effects of APs would be different depending on whether the exposure to these drugs is acute or chronic. In acute exposure, psychomotor retardation, profound inner restlessness and functional impairment due to a “paralysis of volition in absence of sedation,” has been frequently described. On the other hand, chronic exposure would produce a syndrome commonly known as NIDS, which includes apathy-emotional detachment, lack of energy, dysphoria, reduced drive and initiative and loss of creativity among its symptoms (Lewander 1994).

The incidence of these BAEs has not been established. However, the close relationship between the emotional detachment produced by APs and their therapeutic effect (Kapur et al. 2005) suggests that these BAEs could always be present, with different intensity and functional impact. Both NIDS and emotional detachment reports are also extended to second-generation APs (de Haan et al. 2000, 2003; Lataster et al. 2011; Takeuchi et al. 2013). Although NIDS/emotional detachment has been related to extrapyramidal side effects (Gervin et al. 1999; de Haan et al. 2003; Kim and Byun 2010), it could emerge before motor symptoms appear (de Haan et al. 2000). These BAEs have been strongly correlated with functional impairments and lack of compliance (Awad et al. 1996; de Haan et al. 2004; Fischel et al. 2013) with treatment, both with first- and second-generation APs (Lambert et al. 2003; Awad and Voruganti 2004). In the last years, imaging techniques studies have strongly correlated this BAE with the percentage of D2 occupancy (de Haan et al. 2000, 2003).

*Findings in BDs*: Although this BAE has been extensively studied in schizophrenia, there are virtually no data from patients with BDs. Recently, Moncrieff et al. (2009) published an analysis of comments posted by users in an Internet site (http://www.askapatient.com) regarding their subjective experiences with treatment drugs. The authors found that both older and new APs were especially linked to emotional effects, which included feelings of flattened or numbed emotions, loss of interest and motivation, reduced creativity, and perceived changes in personality. Interestingly, 33.6 % of the reports came from self-defined bipolar patients and 11.7 % from depressive patients. However, as this website recorded opinions from users of these drugs, respondents may have misinterpreted the origin of their symptoms and reported them as produced by the drug and not by their underlying condition.

#### Obsessive–compulsive symptomatology (OCS)

Findings from retrospective (Mahendran et al. 2007) and prospective (Schirmbeck et al. 2013) cohort studies suggest a causal relationship between OCS and treatment with second-generation antipsychotics (SGA), particularly clozapine. Furthermore, a dose–response pattern (Lin et al. 2006; Schirmbeck et al. 2011) and an association between duration of treatment and OCS severity have been reported in SGA-treated schizophrenic subjects (Schirmbeck et al. 2011). Among these patients, the prevalence of OCS was found to be of 28.4 % (Lin et al. 2006). Atypical APs have a preferential occupancy of 5HT2 receptors over D2 (Kapur et al. 1998, 1999, 2006) being this profile of antiserotoninergic properties thought to be the cause of OCS induction (Schirmbeck et al. 2011; 2013; Schirmbeck and Zink 2012,2013). Recent studies suggest that OCD and OCS are associated with greater suicidal risk in patients with schizophrenia (Niendam et al. 2009; DeVylder et al. 2012; Hagen et al. 2013; Szmulewicz et al. 2015a), underlining the importance of this BAE.

*Findings in BDs*: a case report of two bipolar type I patients treated with clozapine documented the development of OCS (Lemke and Bustillo 2013). In another case report, three bipolar type I patients treated with high doses of quetiapine also presented with OCS as a side effect (Stamouli and Lykouras 2006). Similarly, Jonkers and de Haan (2002) reported OCS induction due to olanzapine treatment in a BD type II patient. No studies evaluating the functional impact or effects on suicidality of this BAE in BDs patients have been published so far.

#### Decision-making modifications

A recent review on decision-making process in schizophrenia (Adida et al. 2011) found conflicting results. While some studies presented impaired performance on decision-making tests, others did not. Authors stated that a major source of uncontrolled bias was the relationship between AP medication and decision-making performance. For example, neither dosages of APs nor the relationship between performance and percentage of D2 occupancy is recorded in most studies. Current hypotheses state that impairment on decision-making tasks in schizophrenia are related to a biased sensitivity to punishment, which may be due to a motivational deficit as well as an abnormal risk perception (Hutton et al. 2002; Prentice et al. 2005; Heerey et al. 2008; Trémeau et al. 2008). To what extent this may be caused by pathophysiological processes due to the illness itself or to adverse events due to AP treatment is yet to be determined. In line with this, there are some reports of healthy subjects underperforming on the Iowa Gambling Task (IGT) following a sudden dopamine reduction (Sevy et al. 2006; Vrshek-Schallhorn et al. 2013). However, it must be noted that one study assessing IGT performance in de-novo drug-naïve Parkinson’s disease patients (Poletti et al. 2011) found no differences between patients and controls.

*Findings in BDs*: No data regarding this issue in BDs patients were found.

### Lithium

Data from healthy volunteers and case reports have shown that lithium could produce an amotivational syndrome related to its plasma levels. Early studies conducted on healthy volunteers (Schou 1968; Bonetti et al. 1977; Judd et al. 1977; Belmaker et al. 1979; White et al. 1979; Kropf and Muller-Oerlinghausen 1979) found an amotivational syndrome induced by this drug. They also showed that this syndrome had a dose–response pattern, being more prominent in patients with higher lithium serum levels.

Some studies investigated this syndrome in patients with BDs (Bonetti et al. 1977; White et al. 1979; Kropf and Müller-Oerlinghausen 1975; Folstein et al., 1982) finding similar results as in healthy volunteers. Kropf and Müller-Oerlinghausen (1975) conducted a double-blind study on 14 euthymic lithium-treated patients. In one group, subjects maintained their usual blood levels, whereas in the other, subjects had a dose reduction. The results showed that patients with high lithium blood levels were less active and less expansive, however, 29 % of the reduced-blood level group experienced an affective recurrence during follow-up. Whether this syndrome is produced as a side effect of lithium therapy or as consequence of mood stabilization is a matter of controversies. Folstein et al. (1982) compared a group of euthymic BD patients on chronic lithium therapy with normal control subjects using the Visual Analogue Mood Scale for 30 days to evaluate mood variability. They found that patients’ mood was less variable than that of the control group. The authors attributed this unusual degree of mood stability to the effects of lithium treatment and suggested that euthymic patients might view this change as an undesirable aspect of lithium therapy.

### Antiepileptics

#### Lamotrigine

OCS induced by lamotrigine has been described in epileptic (Lombroso 1999; Verma et al. 1999; Lee et al. 2011) and schizophrenic patients (Szmulewicz et al. 2015b). These symptoms appear to be dose-related (over 100 mg/day are described in the above reports) and reversible after treatment discontinuation. Lamotrigine would increase dopamine levels in cortico-striato-thalamo-cortical circuits due to glutamate agonism. This effect would be related to both OCS generation and beneficial outcomes for behavior and cognition (Pittenger et al. 2011). Finally, there are two case reports of lamotrigine induction of hypersexuality (Grabowska-Grzyb et al. 2006) in epileptic patients.

*Findings in BDs:* OCD and tic disorders due to lamotrigine exposure have also been reported in BD patients (Seemüller et al. 2006; Alkin et al. 2007; Kemp et al. 2007; Kuloglu et al. 2009) (Table 1).

#### Valproic acid (VA)

There are few reports on BAE in patients treated with VA in epileptic population and most of them report unspecific effects (Marson et al. 2007; Shehata et al. 2009).

*Findings in BDs:* No studies assessing the impact of these effects on BD patients have been performed.

## Discussion

This review shows the existence of a remarkable knowledge gap on behavioral and emotional side effects of drugs commonly used in the treatment of BD, both in clinical practice and in research. Indeed, the main limitation of this work concerns the lack of systematicity, which is explained, at least partly, by the non-existence of valid instruments and methodology to assess adverse events and also by the lack of previous reviews on this topic. However, this shortcoming does not imply an absence of evidence. Despite being scant and scattered, the available data are consistent, showing that many commonly used drugs would have the potential to produce behavioral and emotional side effects with clinical and theoretical implications. For example, although lithium and lamotrigine are usually seen as “psychologically clean” drugs, both could produce BAEs. Available studies suggest that lithium may produce an amotivational syndrome—which would be dose dependent—and lamotrigine could produce obsessive–compulsive symptoms. In both cases, incidence and functional impact of these BAEs have not been determined and are currently not included in clinical guidelines.

On the other hand, despite the fact that significant amounts of data provide insights about the capacity of SSRIs to produce BAEs due to modifications in emotional processing in healthy volunteers and unipolar depression patients, there are no data for BD patients. Moreover, this lack of information extends even to unipolar depression patients, for which, in the same way, data about how apathy/emotional-blunting impact on overall outcome and functionality are hardly available.

However, negligence of BAEs is especially impressive in the case of the use of APs in BDs patients. Although an increasing number of people affected by these disorders are being treated with these kinds of drugs (Pillarella et al. 2012; Mauer et al. 2014), the lessons learned from schizophrenia about antipsychotic-related BAEs do not seem to have influenced this clinical field. As shown in this review, there are no studies exploring the subjective and behavioral impact of these drugs and their possible therapeutic and functional consequences on patients with BDs. Nor have there been any studies including instruments specifically designed to explore possible BAEs in the many recent trials of APs done on BDs.

Indeed, the frequency and potential impact of the BAEs extensively described in schizophrenia remain uncertain in BD patients. Nevertheless, there are no reasons to expect the absence of such side effects in BDs. If we take into account that NIDS and neuroleptic dysphoria have been consistently related to motor side effects in schizophrenic patients (Awad et al. 1996; Gervin et al. 1999; de Haan et al. 2003; Kim and Byun 2010), then we should expect a higher vulnerability to these BAEs in BD patients due to their comparatively higher sensitivity to extrapyramidal side effects of APs (Gao et al. 2008).

It is clear that the described situation represents an information vacuum with respect to the data that clinicians and patients need to make their therapeutic decisions rationally. Even today, the BAEs here described are not included in the sources of information on adverse effects most commonly used (i.e., Epocrates©). Furthermore, in order to identify some BAEs, it is particularly necessary that patients know about their potential existence in order to become aware that they may suffer them. Due to its phenomenological characteristics, a side effect like apathy may go unnoticed even for the person who is experiencing it. Likewise, the fact that some drugs commonly used to treat BDs might produce biases or distortions in the decision-making process takes the problem to another level. This is an unexplored field, for which the development of new ways to observe and measure BAEs is needed in order to make these potential effects accessible in such a way that they can be discussed by clinicians and users.

The findings discussed here have clear implications for the design of research on the efficacy of pharmacological treatments for BDs and even for mood disorders in general. It is clear that actions to control these side effects should be routinely taken in clinical trials, especially in the case of those drugs that can cause apathy/emotional flattening. Apathy and emotional blunting have a significant overlap with depressive symptoms in the instruments usually used (Marin et al. 1993). Consequently, the presence of these side effects can decrease the scores on depression scales and hide the presence of this new symptomatology at the same time. On the other hand, although limited, the available data suggest that BAE produced by drugs usually indicated in BDs treatment could produce a significant impact on the functionality and clinical outcome of affected subjects. It is possible to speculate that the functional impact attributed to BAE could be involved in the dissociation between syndromic and functional recovery frequently observed in treatments of these disorders.

Finally, although many different medications have shown efficacy in delaying or avoiding new affective crises, there is no framework able to explain the existent hiatus between the biochemical changes produced by these drugs and their effects at the behavioral level. Many new questions and ways to investigate could be addressed if BAEs were considered, as has been the case in schizophrenia (Kapur 2003). For example, BAEs of these drugs could help to explore whether the mechanisms by which lithium, some antipsychotics, and some anticonvulsants prevent mood episodes are similar or not. In the same way, the consideration of these issues could help to formulate an integrative pathophysiology of BDs. For instance, similarities between neuroleptic-induced dysphoria and dopamine withdrawal syndromes, and agitated depression have led to propose a model to explain mixed states in BDs (Strejilevich et al. 2012).

In conclusion, drug-related BAEs have relevant implications for both clinical and research practice. It is urgent to address this issue, thus solving an ethical problem and certainly leading to an increase in the quality and quantity of information on BAEs. However, most of the available evidence in the literature is just descriptive. Therefore, there is an urgent need for developing questionnaires or scales specifically designed to measure this aspect of pharmacological treatment in mood disorders and BDs in particular. Furthermore, given that we are far from having a complete understanding of the pharmacodynamics of the treatments that we daily use, disregarding BAEs in research hypotheses is simply leaving aside a rich source of information.
